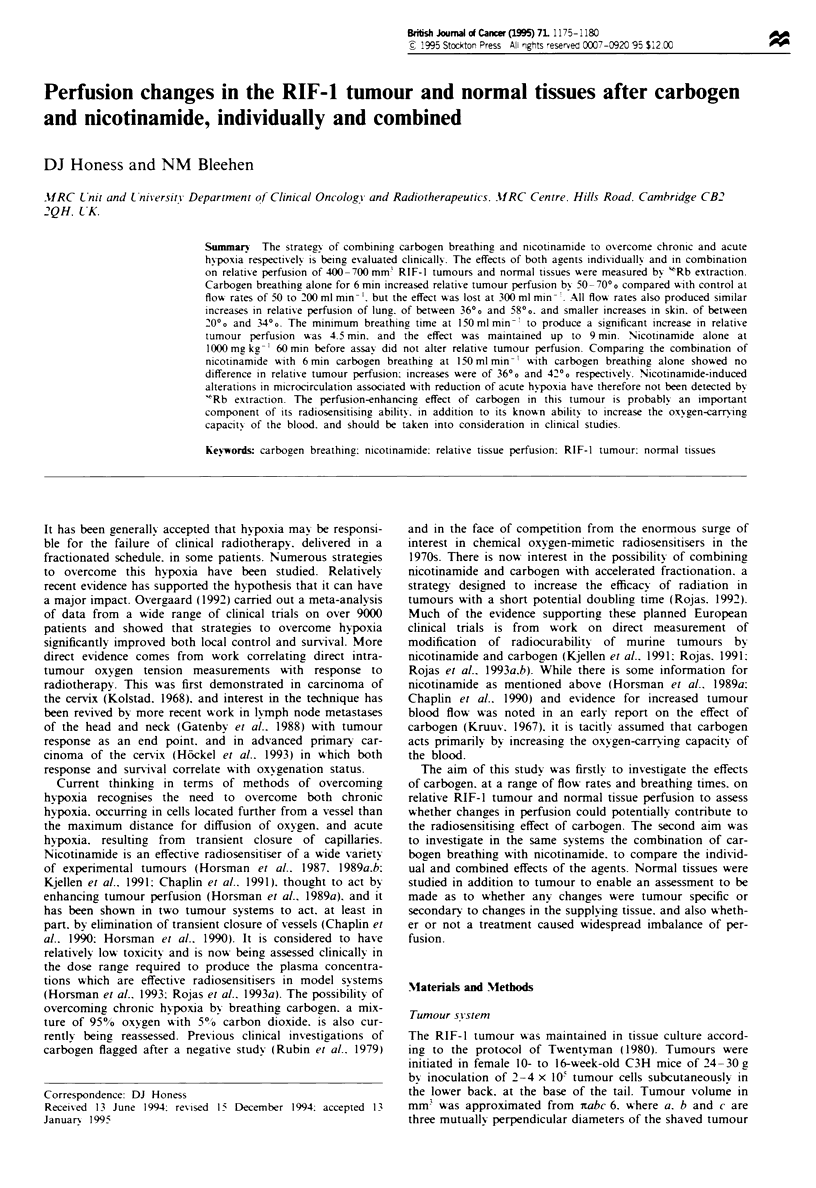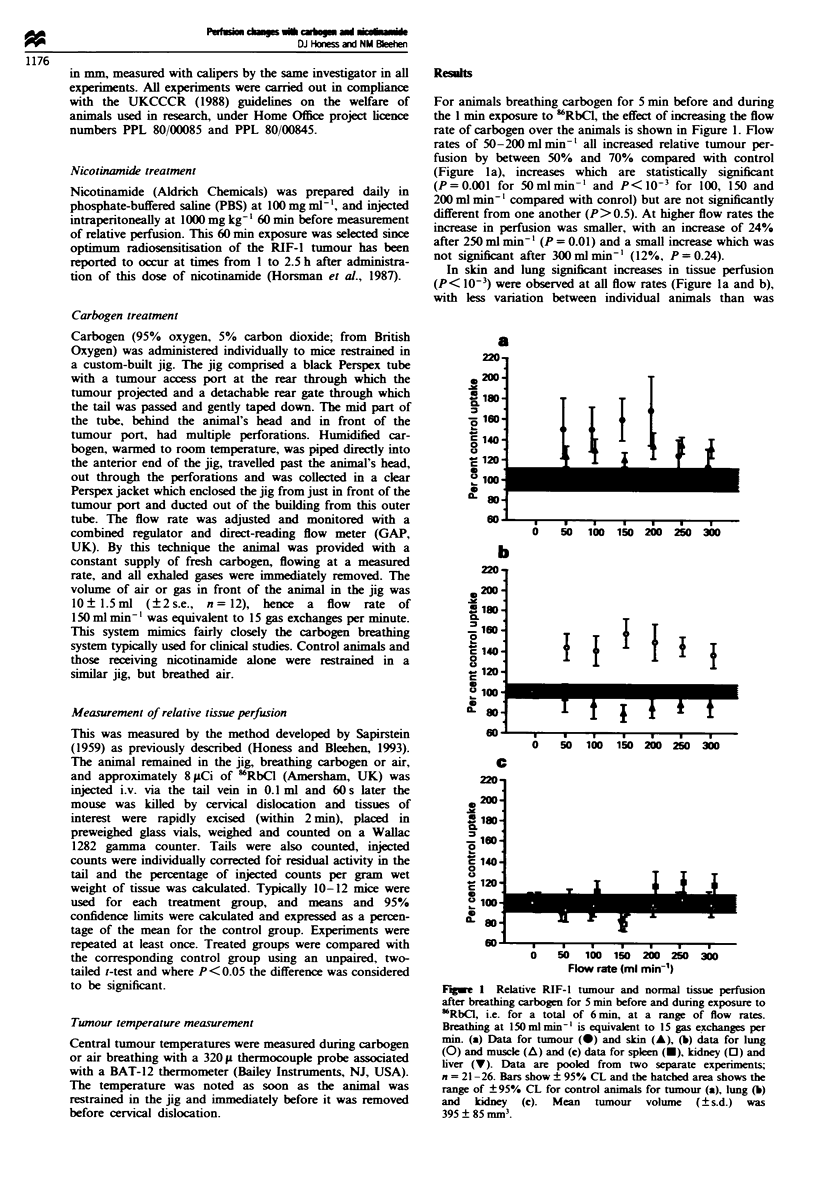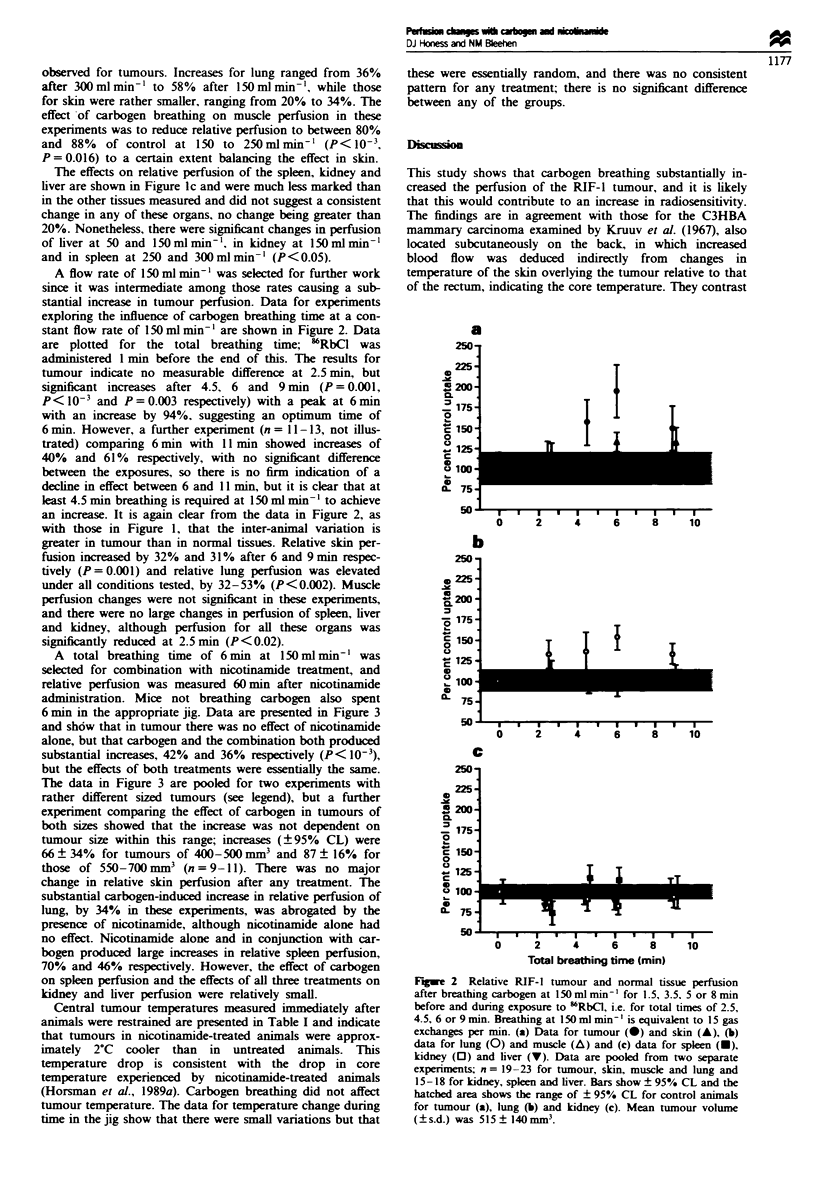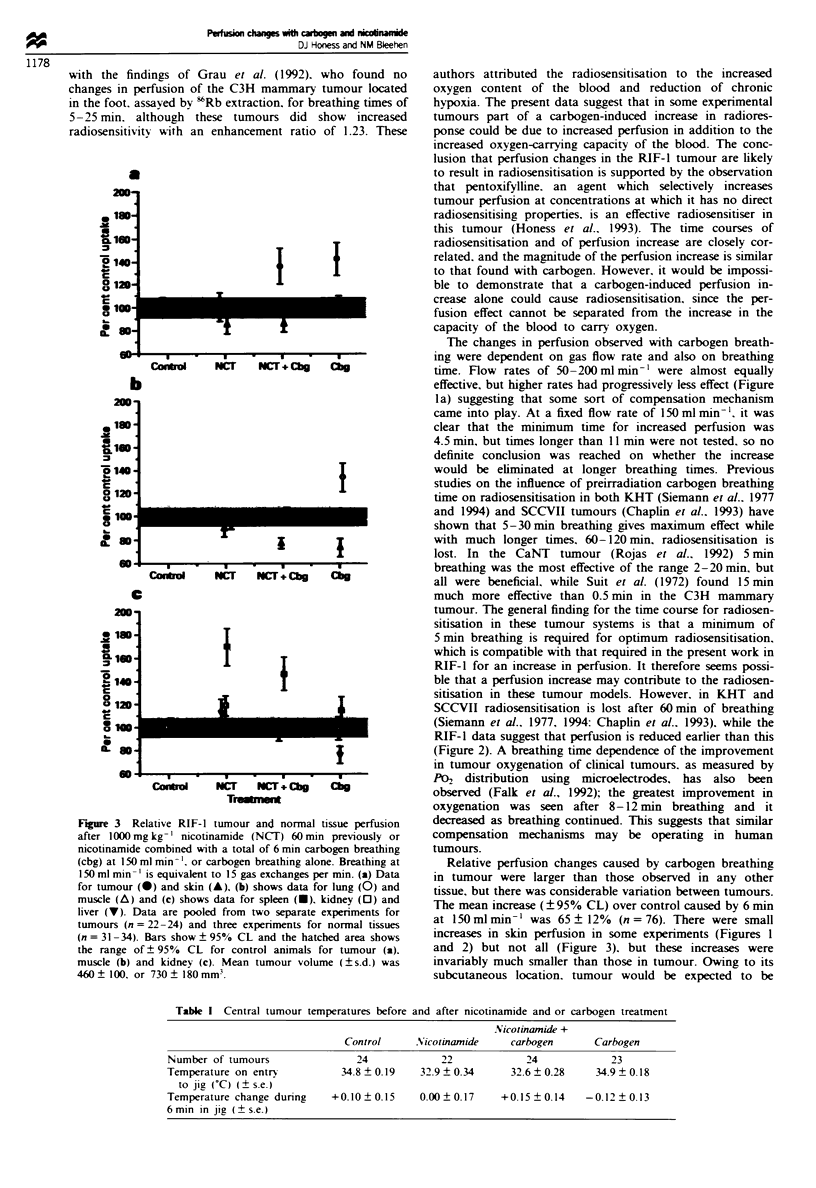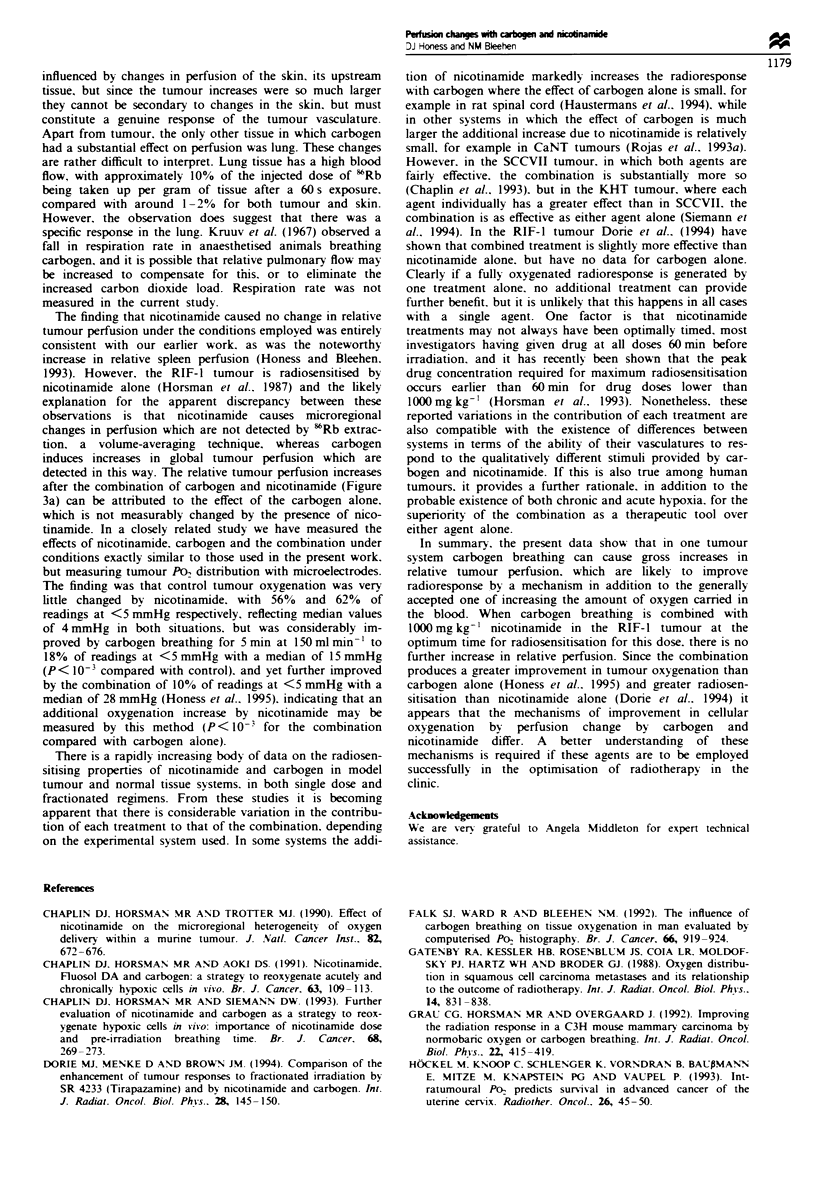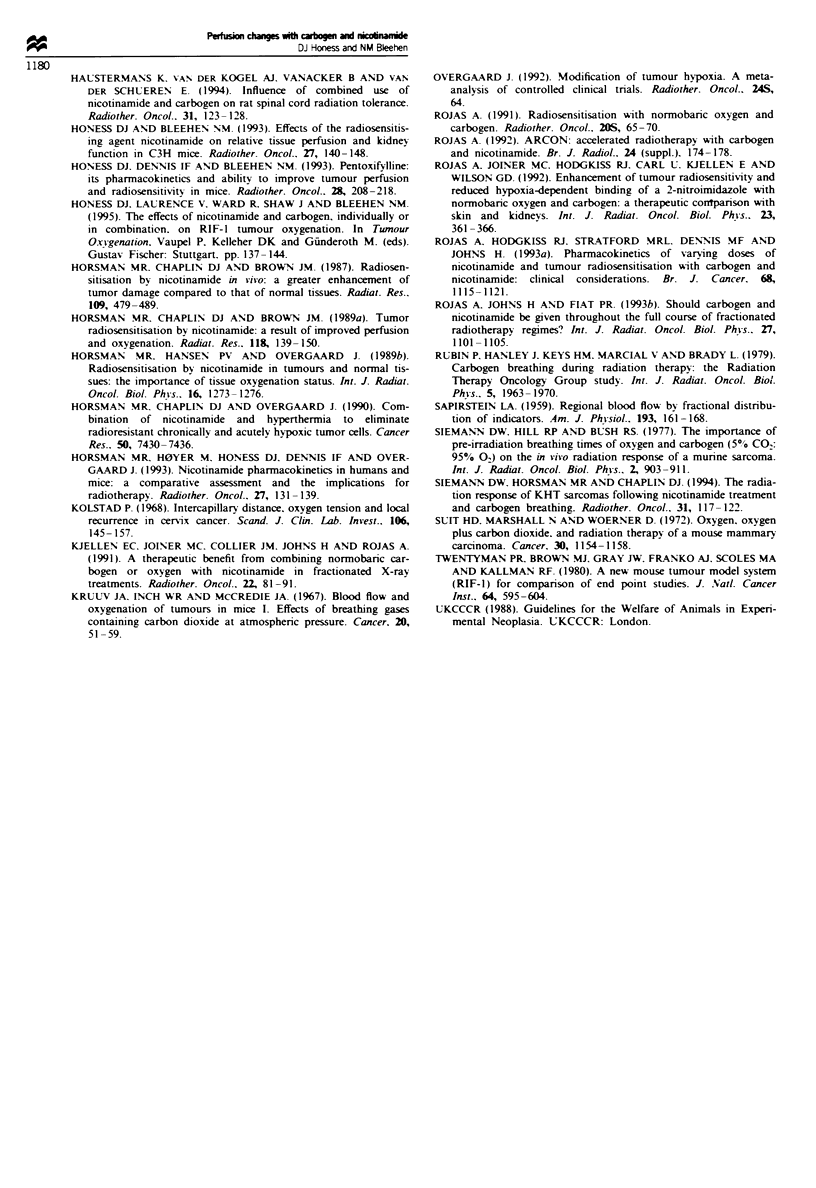# Perfusion changes in the RIF-1 tumour and normal tissues after carbogen and nicotinamide, individually and combined.

**DOI:** 10.1038/bjc.1995.229

**Published:** 1995-06

**Authors:** D. J. Honess, N. M. Bleehen

**Affiliations:** MRC Unit, MRC Centre, Cambridge, UK.

## Abstract

The strategy of combining carbogen breathing and nicotinamide to overcome chronic and acute hypoxia respectively is being evaluated clinically. The effects of both agents individually and in combination on relative perfusion of 400-700 mm3 RIF-1 tumours and normal tissues were measured by 86Rb extraction. Carbogen breathing alone for 6 min increased relative tumour perfusion by 50-70% compared with control at flow rates of 50 to 200 ml min-1, but the effect was lost at 300 ml min-1. All flow rates also produced similar increases in relative perfusion of lung, of between 36% and 58%, and smaller increases in skin, of between 20% and 34%. The minimum breathing time at 150 ml min-1 to produce a significant increase in relative tumour perfusion was 4.5 min, and the effect was maintained up to 9 min. Nicotinamide alone at 1000 mg kg-1 60 min before assay did not alter relative tumour perfusion. Comparing the combination of nicotinamide with 6 min carbogen breathing at 150 ml min-1 with carbogen breathing alone showed no difference in relative tumour perfusion; increases were of 36% and 42% respectively. Nicotinamide-induced alterations in microcirculation associated with reduction of acute hypoxia have therefore not been detected by 86Rb extraction. The perfusion-enhancing effect of carbogen in this tumour is probably an important component of its radiosensitising ability, in addition to its known ability to increase the oxygen-carrying capacity of the blood, and should be taken into consideration in clinical studies.


British Journal of Cancer (1995) 71, 1175–1180

© 1995 Stockton Press All rights reserved 0007–0920/95 $12.00

### Perfusion changes in the RIF-1 tumour and normal tissues after carbogen and nicotinamide, individually and combined

DJ Honess and NM Bleehen

*MRC Unit and University Department of Clinical Oncology and Radiotherapeutics, MRC Centre, Hills Road, Cambridge CB2 2QH, UK.*

**Summary** The strategy of combining carbogen breathing and nicotinamide to overcome chronic and acute hypoxia respectively is being evaluated clinically. The effects of both agents individually and in combination on relative perfusion of 400–700 mm$^3$ RIF-1 tumours and normal tissues were measured by $^{86}$Rb extraction. Carbogen breathing alone for 6 min increased relative tumour perfusion by 50–70% compared with control at flow rates of 50 to 200 ml min$^{-1}$, but the effect was lost at 300 ml min$^{-1}$. All flow rates also produced similar increases in relative perfusion of lung, of between 36% and 58%, and smaller increases in skin, of between 20% and 34%. The minimum breathing time at 150 ml min$^{-1}$ to produce a significant increase in relative tumour perfusion was 4.5 min, and the effect was maintained up to 9 min. Nicotinamide alone at 1000 mg kg$^{-1}$ 60 min before assay did not alter relative tumour perfusion. Comparing the combination of nicotinamide with 6 min carbogen breathing at 150 ml min$^{-1}$ with carbogen breathing alone showed no difference in relative tumour perfusion; increases were of 36% and 42% respectively. Nicotinamide-induced alterations in microcirculation associated with reduction of acute hypoxia have therefore not been detected by $^{86}$Rb extraction. The perfusion-enhancing effect of carbogen in this tumour is probably an important component of its radiosensitising ability, in addition to its known ability to increase the oxygen-carrying capacity of the blood, and should be taken into consideration in clinical studies.

**Keywords:** carbogen breathing; nicotinamide; relative tissue perfusion; RIF-1 tumour; normal tissues

It has been generally accepted that hypoxia may be responsible for the failure of clinical radiotherapy, delivered in a fractionated schedule, in some patients. Numerous strategies to overcome this hypoxia have been studied. Relatively recent evidence has supported the hypothesis that it can have a major impact. Overgaard (1992) carried out a meta-analysis of data from a wide range of clinical trials on over 9000 patients and showed that strategies to overcome hypoxia significantly improved both local control and survival. More direct evidence comes from work correlating direct intratumour oxygen tension measurements with response to radiotherapy. This was first demonstrated in carcinoma of the cervix (Kolstad, 1968), and interest in the technique has been revived by more recent work in lymph node metastases of the head and neck (Gatenby *et al.*, 1988) with tumour response as an end point, and in advanced primary carcinoma of the cervix (Höckel *et al.*, 1993) in which both response and survival correlate with oxygenation status.

Current thinking in terms of methods of overcoming hypoxia recognises the need to overcome both chronic hypoxia, occurring in cells located further from a vessel than the maximum distance for diffusion of oxygen, and acute hypoxia, resulting from transient closure of capillaries. Nicotinamide is an effective radiosensitiser of a wide variety of experimental tumours (Horsman *et al.*, 1987, 1989*a*,*b*; Kjellen *et al.*, 1991; Chaplin *et al.*, 1991), thought to act by enhancing tumour perfusion (Horsman *et al.*, 1989*a*), and it has been shown in two tumour systems to act, at least in part, by elimination of transient closure of vessels (Chaplin *et al.*, 1990; Horsman *et al.*, 1990). It is considered to have relatively low toxicity and is now being assessed clinically in the dose range required to produce the plasma concentrations which are effective radiosensitisers in model systems (Horsman *et al.*, 1993; Rojas *et al.*, 1993*a*). The possibility of overcoming chronic hypoxia by breathing carbogen, a mixture of 95% oxygen with 5% carbon dioxide, is also currently being reassessed. Previous clinical investigations of carbogen flagged after a negative study (Rubin *et al.*, 1979) and in the face of competition from the enormous surge of interest in chemical oxygen-mimetic radiosensitisers in the 1970s. There is now interest in the possibility of combining nicotinamide and carbogen with accelerated fractionation, a strategy designed to increase the efficacy of radiation in tumours with a short potential doubling time (Rojas, 1992). Much of the evidence supporting these planned European clinical trials is from work on direct measurement for modification of radiocurability of murine tumours by nicotinamide and carbogen (Kjellen *et al.*, 1991; Rojas, 1991; Rojas *et al.*, 1993*a*,*b*). While there is some information for nicotinamide as mentioned above (Horsman *et al.*, 1989*a*; Chaplin *et al.*, 1990) and evidence for increased tumour blood flow was noted in an early report on the effect of carbogen (Kruuv, 1967), it is tacitly assumed that carbogen acts primarily by increasing the oxygen-carrying capacity of the blood.

The aim of this study was firstly to investigate the effects of carbogen, at a range of flow rates and breathing times, on relative RIF-1 tumour and normal tissue perfusion to assess whether changes in perfusion could potentially contribute to the radiosensitising effect of carbogen. The second aim was to investigate in the same systems the combination of carbogen breathing with nicotinamide, to compare the individual and combined effects of the agents. Normal tissues were studied in addition to tumour to enable an assessment to be made as to whether any changes were tumour specific or secondary to changes in the supplying tissue, and also whether or not a treatment caused widespread imbalance of perfusion.

### Materials and Methods

### *Tumour system*

The RIF-1 tumour was maintained in tissue culture according to the protocol of Twentyman (1980). Tumours were initiated in female 10- to 16-week-old C3H mice of 24–30 g by inoculation of $2-4 \times 10^5$ tumour cells subcutaneously in the lower back, at the base of the tail. Tumour volume in mm$^3$ was approximated from $\pi abc/6$, where $a$, $b$ and $c$ are three mutually perpendicular diameters of the shaved tumour

Correspondence: DJ Honess
Received 13 June 1994; revised 15 December 1994; accepted 13 January 1995

in mm, measured with calipers by the same investigator in all experiments. All experiments were carried out in compliance with the UKCCCR (1988) guidelines on the welfare of animals used in research, under Home Office project licence numbers PPL 80/00085 and PPL 80/00845.

### *Nicotinamide treatment*

Nicotinamide (Aldrich Chemicals) was prepared daily in phosphate-buffered saline (PBS) at 100 mg $ml^{-1}$, and injected intraperitoneally at 1000 mg $kg^{-1}$ 60 min before measurement of relative perfusion. This 60 min exposure was selected since optimum radiosensitisation of the RIF-1 tumour has been reported to occur at times from 1 to 2.5 h after administration of this dose of nicotinamide (Horsman *et al.*, 1987).

### *Carbogen treatment*

Carbogen (95% oxygen, 5% carbon dioxide; from British Oxygen) was administered individually to mice restrained in a custom-built jig. The jig comprised a black Perspex tube with a tumour access port at the rear through which the tumour projected and a detachable rear gate through which the tail was passed and gently taped down. The mid part of the tube, behind the animal's head and in front of the tumour port, had multiple perforations. Humidified carbogen, warmed to room temperature, was piped directly into the anterior end of the jig, travelled past the animal's head, out through the perforations and was collected in a clear Perspex jacket which enclosed the jig from just in front of the tumour port and ducted out of the building from this outer tube. The flow rate was adjusted and monitored with a combined regulator and direct-reading flow meter (GAP, UK). By this technique the animal was provided with a constant supply of fresh carbogen, flowing at a measured rate, and all exhaled gases were immediately removed. The volume of air or gas in front of the animal in the jig was $10 \pm 1.5$ ml ($\pm 2$ s.e., $n = 12$), hence a flow rate of 150 ml $min^{-1}$ was equivalent to 15 gas exchanges per minute. This system mimics fairly closely the carbogen breathing system typically used for clinical studies. Control animals and those receiving nicotinamide alone were restrained in a similar jig, but breathed air.

### *Measurement of relative tissue perfusion*

This was measured by the method developed by Sapirstein (1959) as previously described (Honess and Bleehen, 1993). The animal remained in the jig, breathing carbogen or air, and approximately 8 $\mu$Ci of $^{86}$RbCl (Amersham, UK) was injected i.v. via the tail vein in 0.1 ml and 60 s later the mouse was killed by cervical dislocation and tissues of interest were rapidly excised (within 2 min), placed in preweighed glass vials, weighed and counted on a Wallac 1282 gamma counter. Tails were also counted, injected counts were individually corrected for residual activity in the tail and the percentage of injected counts per gram wet weight of tissue was calculated. Typically 10–12 mice were used for each treatment group, and means and 95% confidence limits were calculated and expressed as a percentage of the mean for the control group. Experiments were repeated at least once. Treated groups were compared with the corresponding control group using an unpaired, two-tailed *t*-test and where $P < 0.05$ the difference was considered to be significant.

### *Tumour temperature measurement*

Central tumour temperatures were measured during carbogen or air breathing with a 320 $\mu$ thermocouple probe associated with a BAT-12 thermometer (Bailey Instruments, NJ, USA). The temperature was noted as soon as the animal was restrained in the jig and immediately before it was removed before cervical dislocation.

### Results

For animals breathing carbogen for 5 min before and during the 1 min exposure to $^{86}$RbCl, the effect of increasing the flow rate of carbogen over the animals is shown in Figure 1. Flow rates of 50–200 ml $min^{-1}$ all increased relative tumour perfusion by between 50% and 70% compared with control (Figure 1a), increases which are statistically significant ($P = 0.001$ for 50 ml $min^{-1}$ and $P < 10^{-3}$ for 100, 150 and 200 ml $min^{-1}$ compared with conrol) but are not significantly different from one another ($P > 0.5$). At higher flow rates the increase in perfusion was smaller, with an increase of 24% after 250 ml $min^{-1}$ ($P = 0.01$) and a small increase which was not significant after 300 ml $min^{-1}$ (12%, $P = 0.24$).

In skin and lung significant increases in tissue perfusion ($P < 10^{-3}$) were observed at all flow rates (Figure 1a and b), with less variation between individual animals than was

**Figure 1** Relative RIF-1 tumour and normal tissue perfusion after breathing carbogen for 5 min before and during exposure to $^{86}$RbCl, i.e. for a total of 6 min, at a range of flow rates. Breathing at 150 ml $min^{-1}$ is equivalent to 15 gas exchanges per min. (**a**) Data for tumour (●) and skin (▲), (**b**) data for lung (○) and muscle (△) and (**c**) data for spleen (■), kidney (□) and liver (▼). Data are pooled from two separate experiments; $n = 21-26$. Bars show $\pm 95\%$ CL and the hatched area shows the range of $\pm 95\%$ CL for control animals for tumour (**a**), lung (**b**) and kidney (**c**). Mean tumour volume ($\pm$s.d.) was $395 \pm 85$ $mm^3$.

observed for tumours. Increases for lung ranged from 36% after 300 ml $min^{-1}$ to 58% after 150 ml $min^{-1}$, while those for skin were rather smaller, ranging from 20% to 34%. The effect of carbogen breathing on muscle perfusion in these experiments was to reduce relative perfusion to between 80% and 88% of control at 150 to 250 ml $min^{-1}$ ($P < 10^{-3}$, $P = 0.016$) to a certain extent balancing the effect in skin.

The effects on relative perfusion of the spleen, kidney and liver are shown in Figure 1c and were much less marked than in the other tissues measured and did not suggest a consistent change in any of these organs, no change being greater than 20%. Nonetheless, there were significant changes in perfusion of liver at 50 and 150 ml $min^{-1}$, in kidney at 150 ml $min^{-1}$ and in spleen at 250 and 300 ml $min^{-1}$ ($P < 0.05$).

A flow rate of 150 ml $min^{-1}$ was selected for further work since it was intermediate among those rates causing a substantial increase in tumour perfusion. Data for experiments exploring the influence of carbogen breathing time at a constant flow rate of 150 ml $min^{-1}$ are shown in Figure 2. Data are plotted for the total breathing time; $^{86}RbCl$ was administered 1 min before the end of this. The results for tumour indicate no measurable difference at 2.5 min, but significant increases after 4.5, 6 and 9 min ($P = 0.001$, $P < 10^{-3}$ and $P = 0.003$ respectively) with a peak at 6 min with an increase by 94%, suggesting an optimum time of 6 min. However, a further experiment ($n = 11-13$, not illustrated) comparing 6 min with 11 min showed increases of 40% and 61% respectively, with no significant difference between the exposures, so there is no firm indication of a decline in effect between 6 and 11 min, but it is clear that at least 4.5 min breathing is required at 150 ml $min^{-1}$ to achieve an increase. It is again clear from the data in Figure 2, as with those in Figure 1, that the inter-animal variation is greater in tumour than in normal tissues. Relative skin perfusion increased by 32% and 31% after 6 and 9 min respectively ($P = 0.001$) and relative lung perfusion was elevated under all conditions tested, by 32–53% ($P < 0.002$). Muscle perfusion changes were not significant in these experiments, and there were no large changes in perfusion of spleen, liver and kidney, although perfusion for all these organs was significantly reduced at 2.5 min ($P < 0.02$).

A total breathing time of 6 min at 150 ml $min^{-1}$ was selected for combination with nicotinamide treatment, and relative perfusion was measured 60 min after nicotinamide administration. Mice not breathing carbogen also spent 6 min in the appropriate jig. Data are presented in Figure 3 and show that in tumour there was no effect of nicotinamide alone, but that carbogen and the combination both produced substantial increases, 42% and 36% respectively ($P < 10^{-3}$), but the effects of both treatments were essentially the same. The data in Figure 3 are pooled for two experiments with rather different sized tumours (see legend), but a further experiment comparing the effect of carbogen in tumours of both sizes showed that the increase was not dependent on tumour size within this range; increases (±95% CL) were 66 ± 34% for tumours of 400–500 $mm^3$ and 87 ± 16% for those of 550–700 $mm^3$ ($n = 9-11$). There was no major change in relative skin perfusion after any treatment. The substantial carbogen-induced increase in relative perfusion of lung, by 34% in these experiments, was abrogated by the presence of nicotinamide, although nicotinamide alone had no effect. Nicotinamide alone and in conjunction with carbogen produced large increases in relative spleen perfusion, 70% and 46% respectively. However, the effect of carbogen on spleen perfusion and the effects of all three treatments on kidney and liver perfusion were relatively small.

Central tumour temperatures measured immediately after animals were restrained are presented in Table I and indicate that tumours in nicotinamide-treated animals were approximately 2°C cooler than in untreated animals. This temperature drop is consistent with the drop in core temperature experienced by nicotinamide-treated animals (Horsman *et al.*, 1989*a*). Carbogen breathing did not affect tumour temperature. The data for temperature change during time in the jig show that there were small variations but that these were essentially random, and there was no consistent pattern for any treatment; there is no significant difference between any of the groups.

### Discussion

This study shows that carbogen breathing substantially increased the perfusion of the RIF-1 tumour, and it is likely that this would contribute to an increase in radiosensitivity. The findings are in agreement with those for the C3HBA mammary carcinoma examined by Kruuv *et al.* (1967), also located subcutaneously on the back, in which increased blood flow was deduced indirectly from changes in temperature of the skin overlying the tumour relative to that of the rectum, indicating the core temperature. They contrast

**Figure 2** Relative RIF-1 tumour and normal tissue perfusion after breathing carbogen at 150 ml $min^{-1}$ for 1.5, 3.5, 5 or 8 min before and during exposure to $^{86}RbCl$, i.e. for total times of 2.5, 4.5, 6 or 9 min. Breathing at 150 ml $min^{-1}$ is equivalent to 15 gas exchanges per min. (**a**) Data for tumour (●) and skin (▲), (**b**) data for lung (○) and muscle (△) and (**c**) data for spleen (■), kidney (□) and liver (▼). Data are pooled from two separate experiments; $n = 19-23$ for tumour, skin, muscle and lung and 15–18 for kidney, spleen and liver. Bars show ± 95% CL and the hatched area shows the range of ± 95% CL for control animals for tumour (**a**), lung (**b**) and kidney (**c**). Mean tumour volume (±s.d.) was 515 ± 140 $mm^3$.

with the findings of Grau *et al.* (1992), who found no changes in perfusion of the C3H mammary tumour located in the foot, assayed by $^{86}$Rb extraction, for breathing times of 5-25 min, although these tumours did show increased radiosensitivity with an enhancement ratio of 1.23. These authors attributed the radiosensitisation to the increased oxygen content of the blood and reduction of chronic hypoxia. The present data suggest that in some experimental tumours part of a carbogen-induced increase in radioresponse could be due to increased perfusion in addition to the increased oxygen-carrying capacity of the blood. The conclusion that perfusion changes in the RIF-1 tumour are likely to result in radiosensitisation is supported by the observation that pentoxifylline, an agent which selectively increases tumour perfusion at concentrations at which it has no direct radiosensitising properties, is an effective radiosensitiser in this tumour (Honess *et al.*, 1993). The time courses of radiosensitisation and of perfusion increase are closely correlated, and the magnitude of the perfusion increase is similar to that found with carbogen. However, it would be impossible to demonstrate that a carbogen-induced perfusion increase alone could cause radiosensitisation, since the perfusion effect cannot be separated from the increase in the capacity of the blood to carry oxygen.

**Figure 3** Relative RIF-1 tumour and normal tissue perfusion after 1000 mg kg$^{-1}$ nicotinamide (NCT) 60 min previously or nicotinamide combined with a total of 6 min carbogen breathing (cbg) at 150 ml min$^{-1}$, or carbogen breathing alone. Breathing at 150 ml min$^{-1}$ is equivalent to 15 gas exchanges per min. (**a**) Data for tumour (●) and skin (▲), (**b**) shows data for lung (○) and muscle (△) and (**c**) shows data for spleen (■), kidney (□) and liver (▼). Data are pooled from two separate experiments for tumours ($n$ = 22-24) and three experiments for normal tissues ($n$ = 31-34). Bars show ± 95% CL and the hatched area shows the range of ± 95% CL for control animals for tumour (**a**), muscle (**b**) and kidney (**c**). Mean tumour volume (±s.d.) was 460 ± 100, or 730 ± 180 mm$^3$.

The changes in perfusion observed with carbogen breathing were dependent on gas flow rate and also on breathing time. Flow rates of 50-200 ml min$^{-1}$ were almost equally effective, but higher rates had progressively less effect (Figure 1a) suggesting that some sort of compensation mechanism came into play. At a fixed flow rate of 150 ml min$^{-1}$, it was clear that the minimum time for increased perfusion was 4.5 min, but times longer than 11 min were not tested, so no definite conclusion was reached on whether the increase would be eliminated at longer breathing times. Previous studies on the influence of preirradiation carbogen breathing time on radiosensitisation in both KHT (Siemann *et al.*, 1977 and 1994) and SCCVII tumours (Chaplin *et al.*, 1993) have shown that 5-30 min breathing gives maximum effect while with much longer times, 60-120 min, radiosensitisation is lost. In the CaNT tumour (Rojas *et al.*, 1992) 5 min breathing was the most effective of the range 2-20 min, but all were beneficial, while Suit *et al.* (1972) found 15 min much more effective than 0.5 min in the C3H mammary tumour. The general finding for the time course for radiosensitisation in these tumour systems is that a minimum of 5 min breathing is required for optimum radiosensitisation, which is compatible with that required in the present work in RIF-1 for an increase in perfusion. It therefore seems possible that a perfusion increase may contribute to the radiosensitisation in these tumour models. However, in KHT and SCCVII radiosensitisation is lost after 60 min of breathing (Siemann *et al.*, 1977, 1994; Chaplin *et al.*, 1993), while the RIF-1 data suggest that perfusion is reduced earlier than this (Figure 2). A breathing time dependence of the improvement in tumour oxygenation of clinical tumours, as measured by $P_{O_2}$ distribution using microelectrodes, has also been observed (Falk *et al.*, 1992); the greatest improvement in oxygenation was seen after 8-12 min breathing and it decreased as breathing continued. This suggests that similar compensation mechanisms may be operating in human tumours.

Relative perfusion changes caused by carbogen breathing in tumour were larger than those observed in any other tissue, but there was considerable variation between tumours. The mean increase (±95% CL) over control caused by 6 min at 150 ml min$^{-1}$ was 65 ± 12% ($n$ = 76). There were small increases in skin perfusion in some experiments (Figures 1 and 2) but not all (Figure 3), but these increases were invariably much smaller than those in tumour. Owing to its subcutaneous location, tumour would be expected to be

**Table I** Central tumour temperatures before and after nicotinamide and or carbogen treatment

| | Control | Nicotinamide | Nicotinamide + carbogen | Carbogen |
|---|---|---|---|---|
| Number of tumours | 24 | 22 | 24 | 23 |
| Temperature on entry to jig (°C) (± s.e.) | 34.8 ± 0.19 | 32.9 ± 0.34 | 32.6 ± 0.28 | 34.9 ± 0.18 |
| Temperature change during 6 min in jig (± s.e.) | +0.10 ± 0.15 | 0.00 ± 0.17 | +0.15 ± 0.14 | −0.12 ± 0.13 |

influenced by changes in perfusion of the skin, its upstream tissue, but since the tumour increases were so much larger they cannot be secondary to changes in the skin, but must constitute a genuine response of the tumour vasculature. Apart from tumour, the only other tissue in which carbogen had a substantial effect on perfusion was lung. These changes are rather difficult to interpret. Lung tissue has a high blood flow, with approximately 10% of the injected dose of $^{86}$Rb being taken up per gram of tissue after a 60 s exposure, compared with around 1–2% for both tumour and skin. However, the observation does suggest that there was a specific response in the lung. Kruuv *et al.* (1967) observed a fall in respiration rate in anaesthetised animals breathing carbogen, and it is possible that relative pulmonary flow may be increased to compensate for this, or to eliminate the increased carbon dioxide load. Respiration rate was not measured in the current study.

The finding that nicotinamide caused no change in relative tumour perfusion under the conditions employed was entirely consistent with our earlier work, as was the noteworthy increase in relative spleen perfusion (Honess and Bleehen, 1993). However, the RIF-1 tumour is radiosensitised by nicotinamide alone (Horsman *et al.*, 1987) and the likely explanation for the apparent discrepancy between these observations is that nicotinamide causes microregional changes in perfusion which are not detected by $^{86}$Rb extraction, a volume-averaging technique, whereas carbogen induces increases in global tumour perfusion which are detected in this way. The relative tumour perfusion increases after the combination of carbogen and nicotinamide (Figure 3a) can be attributed to the effect of the carbogen alone, which is not measurably changed by the presence of nicotinamide. In a closely related study we have measured the effects of nicotinamide, carbogen and the combination under conditions exactly similar to those used in the present work, but measuring tumour $P\text{O}_2$ distribution with microelectrodes. The finding was that control tumour oxygenation was very little changed by nicotinamide, with 56% and 62% of readings at <5 mmHg respectively, reflecting median values of 4 mmHg in both situations, but was considerably improved by carbogen breathing for 5 min at 150 ml min$^{-1}$ to 18% of readings at <5 mmHg with a median of 15 mmHg ($P < 10^{-3}$ compared with control), and yet further improved by the combination of 10% of readings at <5 mmHg with a median of 28 mmHg (Honess *et al.*, 1995), indicating that an additional oxygenation increase by nicotinamide may be measured by this method ($P < 10^{-3}$ for the combination compared with carbogen alone).

There is a rapidly increasing body of data on the radiosensitising properties of nicotinamide and carbogen in model tumour and normal tissue systems, in both single dose and fractionated regimens. From these studies it is becoming apparent that there is considerable variation in the contribution of each treatment to that of the combination, depending on the experimental system used. In some systems the addition of nicotinamide markedly increases the radioresponse with carbogen where the effect of carbogen alone is small, for example in rat spinal cord (Haustermans *et al.*, 1994), while in other systems in which the effect of carbogen is much larger the additional increase due to nicotinamide is relatively small, for example in CaNT tumours (Rojas *et al.*, 1993*a*). However, in the SCCVII tumour, in which both agents are fairly effective, the combination is substantially more so (Chaplin *et al.*, 1993), but in the KHT tumour, where each agent individually has a greater effect than in SCCVII, the combination is as effective as either agent alone (Siemann *et al.*, 1994). In the RIF-1 tumour Dorie *et al.*, (1994) have shown that combined treatment is slightly more effective than nicotinamide alone, but have no data for carbogen alone. Clearly if a fully oxygenated radioresponse is generated by one treatment alone, no additional treatment can provide further benefit, but it is unlikely that this happens in all cases with a single agent. One factor is that nicotinamide treatments may not always have been optimally timed, most investigators having given drug at all doses 60 min before irradiation, and it has recently been shown that the peak drug concentration required for maximum radiosensitisation occurs earlier than 60 min for drug doses lower than 1000 mg kg$^{-1}$ (Horsman *et al.*, 1993). Nonetheless, these reported variations in the contribution of each treatment are also compatible with the existence of differences between systems in terms of the ability of their vasculatures to respond to the qualitatively different stimuli provided by carbogen and nicotinamide. If this is also true among human tumours, it provides a further rationale, in addition to the probable existence of both chronic and acute hypoxia, for the superiority of the combination as a therapeutic tool over either agent alone.

In summary, the present data show that in one tumour system carbogen breathing can cause gross increases in relative tumour perfusion, which are likely to improve radioresponse by a mechanism in addition to the generally accepted one of increasing the amount of oxygen carried in the blood. When carbogen breathing is combined with 1000 mg kg$^{-1}$ nicotinamide in the RIF-1 tumour at the optimum time for radiosensitisation for this dose, there is no further increase in relative perfusion. Since the combination produces a greater improvement in tumour oxygenation than carbogen alone (Honess *et al.*, 1995) and greater radiosensitisation than nicotinamide alone (Dorie *et al.*, 1994) it appears that the mechanisms of improvement in cellular oxygenation by perfusion change by carbogen and nicotinamide differ. A better understanding of these mechanisms is required if these agents are to be employed successfully in the optimisation of radiotherapy in the clinic.

**Acknowledgements**
We are very grateful to Angela Middleton for expert technical assistance.

**References**

CHAPLIN DJ, HORSMAN MR AND TROTTER MJ. (1990). Effect of nicotinamide on the microregional heterogeneity of oxygen delivery within a murine tumour. *J. Natl. Cancer Inst.*, **82**, 672–676.

CHAPLIN DJ, HORSMAN MR AND AOKI DS. (1991). Nicotinamide, Fluosol DA and carbogen: a strategy to reoxygenate acutely and chronically hypoxic cells *in vivo*. *Br. J. Cancer*, **63**, 109–113.

CHAPLIN DJ, HORSMAN MR AND SIEMANN DW. (1993). Further evaluation of nicotinamide and carbogen as a strategy to reoxygenate hypoxic cells *in vivo*: importance of nicotinamide dose and pre-irradiation breathing time. *Br. J. Cancer*, **68**, 269–273.

DORIE MJ, MENKE D AND BROWN JM. (1994). Comparison of the enhancement of tumour responses to fractionated irradiation by SR 4233 (Tirapazamine) and by nicotinamide and carbogen. *Int. J. Radiat. Oncol. Biol. Phys.*, **28**, 145–150.

FALK SJ, WARD R AND BLEEHEN NM. (1992). The influence of carbogen breathing on tissue oxygenation in man evaluated by computerised $P\text{O}_2$ histography. *Br. J. Cancer*, **66**, 919–924.

GATENBY RA, KESSLER HB, ROSENBLUM JS, COIA LR, MOLDOFSKY PJ, HARTZ WH AND BRODER GJ. (1988). Oxygen distribution in squamous cell carcinoma metastases and its relationship to the outcome of radiotherapy. *Int. J. Radiat. Oncol. Biol. Phys.*, **14**, 831–838.

GRAU CG, HORSMAN MR AND OVERGAARD J. (1992). Improving the radiation response in a C3H mouse mammary carcinoma by normobaric oxygen or carbogen breathing. *Int. J. Radiat. Oncol. Biol. Phys.*, **22**, 415–419.

HÖCKEL M, KNOOP C, SCHLENGER K, VORNDRAN B, BAUßMANN E, MITZE M, KNAPSTEIN PG AND VAUPEL P. (1993). Intratumoural $P\text{O}_2$ predicts survival in advanced cancer of the uterine cervix. *Radiother. Oncol.*, **26**, 45–50.

HAUSTERMANS K, VAN DER KOGEL AJ, VANACKER B AND VAN DER SCHUEREN E. (1994). Influence of combined use of nicotinamide and carbogen on rat spinal cord radiation tolerance. *Radiother. Oncol.*, **31**, 123-128.

HONESS DJ AND BLEEHEN NM. (1993). Effects of the radiosensitising agent nicotinamide on relative tissue perfusion and kidney function in C3H mice. *Radiother. Oncol.*, **27**, 140-148.

HONESS DJ, DENNIS IF AND BLEEHEN NM. (1993). Pentoxifylline: its pharmacokinetics and ability to improve tumour perfusion and radiosensitivity in mice. *Radiother. Oncol.*, **28**, 208-218.

HONESS DJ, LAURENCE V, WARD R, SHAW J AND BLEEHEN NM. (1995). The effects of nicotinamide and carbogen, individually or in combination, on RIF-1 tumour oxygenation. In *Tumour Oxygenation*, Vaupel P, Kelleher DK and Günderoth M. (eds). Gustav Fischer: Stuttgart, pp. 137-144.

HORSMAN MR, CHAPLIN DJ AND BROWN JM. (1987). Radiosensitisation by nicotinamide *in vivo*: a greater enhancement of tumor damage compared to that of normal tissues. *Radiat. Res.*, **109**, 479-489.

HORSMAN MR, CHAPLIN DJ AND BROWN JM. (1989*a*). Tumor radiosensitisation by nicotinamide: a result of improved perfusion and oxygenation. *Radiat. Res.*, **118**, 139-150.

HORSMAN MR, HANSEN PV AND OVERGAARD J. (1989*b*). Radiosensitisation by nicotinamide in tumours and normal tissues: the importance of tissue oxygenation status. *Int. J. Radiat. Oncol. Biol. Phys.*, **16**, 1273-1276.

HORSMAN MR, CHAPLIN DJ AND OVERGAARD J. (1990). Combination of nicotinamide and hyperthermia to eliminate radioresistant chronically and acutely hypoxic tumor cells. *Cancer Res.*, **50**, 7430-7436.

HORSMAN MR, HØYER M, HONESS DJ, DENNIS IF AND OVERGAARD J. (1993). Nicotinamide pharmacokinetics in humans and mice: a comparative assessment and the implications for radiotherapy. *Radiother. Oncol.*, **27**, 131-139.

KOLSTAD P. (1968). Intercapillary distance, oxygen tension and local recurrence in cervix cancer. *Scand. J. Clin. Lab. Invest.*, **106**, 145-157.

KJELLEN EC, JOINER MC, COLLIER JM, JOHNS H AND ROJAS A. (1991). A therapeutic benefit from combining normobaric carbogen or oxygen with nicotinamide in fractionated X-ray treatments. *Radiother. Oncol.*, **22**, 81-91.

KRUUV JA, INCH WR AND MCCREDIE JA. (1967). Blood flow and oxygenation of tumours in mice I. Effects of breathing gases containing carbon dioxide at atmospheric pressure. *Cancer*, **20**, 51-59.

OVERGAARD J. (1992). Modification of tumour hypoxia. A meta-analysis of controlled clinical trials. *Radiother. Oncol.*, **24S**, 64.

ROJAS A. (1991). Radiosensitisation with normobaric oxygen and carbogen. *Radiother. Oncol.*, **20S**, 65-70.

ROJAS A. (1992). ARCON: accelerated radiotherapy with carbogen and nicotinamide. *Br. J. Radiol.*, **24** (suppl.), 174-178.

ROJAS A, JOINER MC, HODGKISS RJ, CARL U, KJELLEN E AND WILSON GD. (1992). Enhancement of tumour radiosensitivity and reduced hypoxia-dependent binding of a 2-nitroimidazole with normobaric oxygen and carbogen: a therapeutic comparison with skin and kidneys. *Int. J. Radiat. Oncol. Biol. Phys.*, **23**, 361-366.

ROJAS A, HODGKISS RJ, STRATFORD MRL, DENNIS MF AND JOHNS H. (1993*a*). Pharmacokinetics of varying doses of nicotinamide and tumour radiosensitisation with carbogen and nicotinamide: clinical considerations. *Br. J. Cancer*, **68**, 1115-1121.

ROJAS A, JOHNS H AND FIAT PR. (1993*b*). Should carbogen and nicotinamide be given throughout the full course of fractionated radiotherapy regimes? *Int. J. Radiat. Oncol. Biol. Phys.*, **27**, 1101-1105.

RUBIN P, HANLEY J, KEYS HM, MARCIAL V AND BRADY L. (1979). Carbogen breathing during radiation therapy: the Radiation Therapy Oncology Group study. *Int. J. Radiat. Oncol. Biol. Phys.*, **5**, 1963-1970.

SAPIRSTEIN LA. (1959). Regional blood flow by fractional distribution of indicators. *Am. J. Physiol.*, **193**, 161-168.

SIEMANN DW, HILL RP AND BUSH RS. (1977). The importance of pre-irradiation breathing times of oxygen and carbogen (5% $CO_2$: 95% $O_2$) on the *in vivo* radiation response of a murine sarcoma. *Int. J. Radiat. Oncol. Biol. Phys.*, **2**, 903-911.

SIEMANN DW, HORSMAN MR AND CHAPLIN DJ. (1994). The radiation response of KHT sarcomas following nicotinamide treatment and carbogen breathing. *Radiother. Oncol.*, **31**, 117-122.

SUIT HD, MARSHALL N AND WOERNER D. (1972). Oxygen, oxygen plus carbon dioxide, and radiation therapy of a mouse mammary carcinoma. *Cancer*, **30**, 1154-1158.

TWENTYMAN PR, BROWN MJ, GRAY JW, FRANKO AJ, SCOLES MA AND KALLMAN RF. (1980). A new mouse tumour model system (RIF-1) for comparison of end point studies. *J. Natl. Cancer Inst.*, **64**, 595-604.

UKCCCR (1988). Guidelines for the Welfare of Animals in Experimental Neoplasia. UKCCCR: London.